# Longitudinal TSPO expression in tau transgenic P301S mice predicts increased tau accumulation and deteriorated spatial learning

**DOI:** 10.1186/s12974-020-01883-5

**Published:** 2020-07-13

**Authors:** Florian Eckenweber, Jose Medina-Luque, Tanja Blume, Christian Sacher, Gloria Biechele, Karin Wind, Maximilian Deussing, Nils Briel, Simon Lindner, Guido Boening, Barbara von Ungern-Sternberg, Marcus Unterrainer, Nathalie L. Albert, Andreas Zwergal, Johannes Levin, Peter Bartenstein, Paul Cumming, Axel Rominger, Günter U. Höglinger, Jochen Herms, Matthias Brendel

**Affiliations:** 1 Department of Nuclear Medicine, University Hospital of Munich, LMU Munich, 81377 Munich, Germany; 2 Center of Neuropathology and Prion Research, University Hospital of Munich, LMU Munich, 81377 Munich, Germany; 3 German Center for Neurodegenerative Diseases (DZNE) Munich, 81377 Munich, Germany; 4 German Center for Vertigo and Balance Disorders, DSGZ, University Hospital of Munich, LMU Munich, 81377 Munich, Germany; 5 Department of Neurology, University Hospital of Munich, LMU Munich, 81377 Munich, Germany; 6 Munich Cluster for Systems Neurology (SyNergy), 81377 Munich, Germany; 7 Department of Nuclear Medicine, Inselspital Bern, Bern, Switzerland; 8 School of Psychology and Counselling and IHBI, Queensland University of Technology, Brisbane, Australia; 9 Department of Neurology, Hannover Medical School, Hannover, Germany; 10 Department of Neurology, Technical University of Munich, Munich, Germany

**Keywords:** TSPO, PET, Tau, Microglia, P301S, Spatial learning, Glucose metabolism

## Abstract

**Background:**

P301S tau transgenic mice show age-dependent accumulation of neurofibrillary tangles in the brainstem, hippocampus, and neocortex, leading to neuronal loss and cognitive deterioration. However, there is hitherto only sparse documentation of the role of neuroinflammation in tau mouse models. Thus, we analyzed longitudinal microglial activation by small animal 18 kDa translocator protein positron-emission-tomography (TSPO μPET) imaging in vivo, in conjunction with terminal assessment of tau pathology, spatial learning, and cerebral glucose metabolism.

**Methods:**

Transgenic P301S (*n* = 33) and wild-type (*n* = 18) female mice were imaged by ^18^F-GE-180 TSPO μPET at the ages of 1.9, 3.9, and 6.4 months. We conducted behavioral testing in the Morris water maze, ^18^F-fluordesoxyglucose (^18^F-FDG) μPET, and AT8 tau immunohistochemistry at 6.3–6.7 months. Terminal microglial immunohistochemistry served for validation of TSPO μPET results in vivo, applying target regions in the brainstem, cortex, cerebellum, and hippocampus. We compared the results with our historical data in amyloid-β mouse models.

**Results:**

TSPO expression in all target regions of P301S mice increased exponentially from 1.9 to 6.4 months, leading to significant differences in the contrasts with wild-type mice at 6.4 months (+ 11–23%, all *p* < 0.001), but the apparent microgliosis proceeded more slowly than in our experience in amyloid-β mouse models. Spatial learning and glucose metabolism of AT8-positive P301S mice were significantly impaired at 6.3–6.5 months compared to the wild-type group. Longitudinal increases in TSPO expression predicted greater tau accumulation and lesser spatial learning performance at 6.3–6.7 months.

**Conclusions:**

Monitoring of TSPO expression as a surrogate of microglial activation in P301S tau transgenic mice by μPET indicates a delayed time course when compared to amyloid-β mouse models. Detrimental associations of microglial activation with outcome parameters are opposite to earlier data in amyloid-β mouse models. The contribution of microglial response to pathology accompanying amyloid-β and tau over-expression merits further investigation.

## Introduction

Along with features such as extracellular accumulation of amyloid-β plaques and neuroinflammation, the intracellular aggregation of misfolded tau protein as neurofibrillary tangles (NFT) constitutes one of the neuropathological hallmarks of Alzheimer disease (AD) [1]. Under physiological circumstances, the microtubule-associated protein tau (MAPT) plays an important role in binding and stabilizing microtubules, regulating axonal transport, interacting with filaments of the cellular cytoskeleton, and probably also contributes to DNA/RNA protection in the nucleus [2]. In AD and non-AD tauopathies, though, the natively soluble and unfolded tau protein undergoes a conformational change via mechanisms such as hyperphosphorylation and misfolding, leading to diminished physiological functions of tau and its accumulation as NFT [3–5]. Deposition of hyperphosphorylated tau in brain is associated with neuroinflammation [5], which may exacerbate the ongoing tauopathy and amyloid-β accumulation, while aggravating neuronal degeneration [4, 5]. Indeed, the neuroinflammation in AD shows spatial overlap with deposition of amyloid-β and NFT accumulation [6]. Furthermore, particular components of the neuroinflammatory cascade promote the development of NFT [7]. Importantly, the onset of neuroinflammation occurs early in tauopathies, suggesting that biomarkers of neuroinflammation might serve as a tool to predict the individual disease course [8].

The transgenic P301S mouse model accumulates tau in the brainstem [9–11], hippocampus [10, 12], and cerebral cortex [10], and this accumulation is accompanied by a decline in spatial learning [12]. Immunohistochemical (IHC) analysis revealed increased microglial activation in transgenic P301S mice at 5 months of age, compared to findings in wild-type mice [13]. Other studies demonstrate the capacity of wild-type mouse microglia to phagocytize NFTs accumulating in the brain of P301S mice [14] and likewise in cultured neurons from P301S mice [15], consistent with a dual role of microglial activation in exerting neuroprotective [14] and neurodegenerative effects [15]. However, the time course of microglial neuroinflammation and its net effect on neurodegeneration is not yet established in this mouse model of tauopathy.

Because understanding the role of neuroinflammation in AD and non-AD tauopathies is of crucial importance, we undertook longitudinal monitoring of microglial activation in P301S mice by means of ^18^F-GE-180 μPET in vivo, extending a technique we have established in amyloid-β mouse models. The tracer ^18^F-GE-180 binds to the 18 kDa translocator protein (TSPO) expressed in activated microglial cells in living mouse brain, showing excellent correlation with ex vivo validation in several different amyloid-β mouse models [16–19]. We now aimed to test the predictive value of early microglial activation in this tau mouse model by undertaking serial ^18^F-GE-180 μPET until 6.4 months of age, augmented by analyses of spatial learning with the Morris water maze test (MWM) and glucose metabolism with ^18^F-fluorodesoxyglucose (^18^F-FDG) μPET. Finally, we made an IHC examination of tau and microglia by AT8, IBA1, and CD68 antisera. Moreover, we compared the temporal kinetics of microglial activation of the tau mouse model with corresponding findings retrieved from our historical amyloid-β mouse model studies.

## Materials and methods

### Animals and study design

**Fig. 1:**
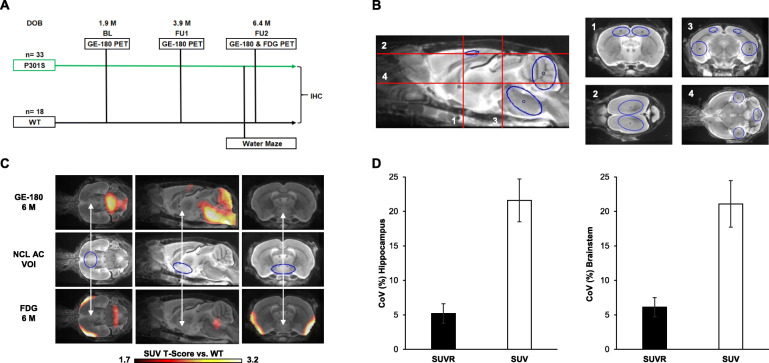
Study design and methodology. **a** Schematic illustration of the study design. TSPO μPET was performed at 1.9, 3.9, and 6.4 months of age and ^18^F-FDG μPET at 6.4 months. Morris water maze was conducted before the final μPET scan. After the final scans, randomly selected mouse brains were processed for immunohistochemistry (IHC) analyses. **b** Target regions used in the study projected on a mouse brain MRI atlas: bilateral cortical (CTX), bilateral hippocampal (HIP), cerebellar (CBL), and brainstem (BRST) VOIs. **c** The middle row shows the bilateral nucleus accumbens (NCL AC) pseudo reference regions projected on mouse brain MRI. Statistical parametric mapping (SPM) shows lacking differences for ^18^F-FDG and^18^F-GE-180 in NAC at 6.4 months of age in SUV-scaled images from P301S mice contrasted against WT mice. **d** Robustness of ^18^F-GE-180 TSPO μPET values in analysis groups (total of six groups of P301S and WT mice) for SUV calculation (white) and pseudo reference region scaling (black) expressed as mean %-CoV (± SD). Error bars indicate SD. BL, baseline; FU, follow-up; SUV, standardized uptake value; SUVR, standardized uptake value ratio; CoV, coefficient of variance

All experiments were performed in compliance with the National Guidelines for Animal Protection, Germany, and with the approval of the regional animal committee (Regierung Oberbayern) and were overseen by a veterinarian. Animals were housed in a temperature- and humidity-controlled environment with 12 h light-dark cycle, with free access to food (Sniff, Soest, Germany) and water. μPET experiments were carried out in homozygous female human tau P301S mice (*n* = 33), a mouse line expressing the human 0N4R tau isoform with the P301S mutation in exon 10 of the MAPT gene under control of the murine thy1 promoter [10], whereas control studies were conducted in age and sex matched wild-type (WT, *n* = 18) mice. TSPO μPET examinations were performed in a longitudinal design at baseline (1.9 months of age) and two follow-up measurements (3.9 and 6.4 months of age) (Fig. 1a). ^18^F-FDG μPET scans were conducted at the age of 6.4–6.5 months. The MWM test was administered at 12 ± 7 days before the final TSPO μPET scan in P301S (*n* = 22) and WT (*n* = 18) mice. After recovery of 2–6 days following the final μPET scans, randomly selected brains from P301S (*n* = 14) mice and WT (*n* = 5) mice were processed for IBA1, CD68, and AT8 IHC in the brainstem and cortex. Additional IHC analyses were conducted in small subgroups (*n* = 3) of P301S mice and (*n* = 2) WT mice at 2.7 and 4.8/4.5 months of age. Mice intended for IHC were deeply anaesthetized prior to transcardial perfusion with saline followed by 4% paraformaldehyde and subsequent brain extraction. Brains were then fixed by immersion in 4% paraformaldehyde at 4 °C for 10 h and then transfered to phosphate buffered saline (PBS). Samples were stored in PBS with 0.01% sodium azide at 4 °C until preparation for staining. Representative 50 μm thick slices per animal were then cut in the axial plane using a vibratome (VT 1000 S, Leica, Wetzlar, Germany). We reprocessed historical μPET ^18^F-GE-180 scans from amyloid-β APP/PS1 [19] and *App*^*NL-G-F*^ mice [20] for comparison of their longitudinal microglial activation with present findings associated with tau accumulation in P301S mice.

### Radiochemistry and μPET Imaging

Radiosynthesis of ^18^F-GE-180 was performed as previously described [16], and ^18^F-FDG was purchased commercially. μPET imaging was described as reported previously [16]. In brief, all mice were anesthetized with isoflurane (1.5%, delivered at 3.5 L/min) and were placed in the aperture of the Siemens Inveon DPET. ^18^F-GE-180 TSPO μPET with an emission window of 60–90 min p.i. was used to measure cerebral TSPO expression, and (on another day) ^18^F-FDG μPET with an emission window of 30–60 min p.i. was used for assessment of cerebral glucose metabolism.

### μPET image analysis

All analyses were performed using PMOD (V3.5, PMOD technologies, Basel, Switzerland). After co-registration to an MRI mouse atlas [17], we conducted intensity normalization of images to standardized uptake value (SUV) relative to uptake in the myocardium for ^18^F-GE-180 μPET [21] and by conventional SUV calculation for ^18^F-FDG μPET. Predefined bilateral cortical (CTX, 24 mm^3^), bilateral hippocampal (HIP, 11 mm^3^), cerebellum (CBL, 12 mm^3^), and brainstem (BRST, 12 mm^3^) target volumes of interest (VOIs) were applied for both tracers (Fig. 1b). We selected these target VOIs based on our immunohistochemical findings of tau accumulation in our earlier studies of the P301S mouse model [9–12].

Voxel-based comparisons of SUV maps between groups of P301S and WT mice were performed using statistical parametric mapping (SPM, described below) to identify first a suitable reference tissue for μPET quantification. Here, this criterion is met by any brain region in which tracer uptake did not differ with genotype or age, either for ^18^F-FDG or for ^18^F-GE-180. Our analysis showed that the bilateral nucleus accumbens (NAC, 10 mm^3^) served adequately as a pseudo reference region for calculation of SUV-ratio (SUVR) values for both μPET tracers (Fig. 1c). We next calculated target-to-reference tissue SUVRs, i.e., SUVR_CTX/NAC_, SUVR_HIP/NAC_, SUVR_CBL/NAC_, and SUVR_BRST/NAC_, for ^18^F-GE-180 and ^18^F-FDG μPET. To quantify longitudinal changes in microglia activation, the percentage change between SUVR at baseline and the last follow-up scan was calculated. To allow longitudinal analysis of combined regions of interest, the area under the curve (AUC) of ^18^F-GE-180 μPET SUVR between baseline and the terminal imaging time point was calculated as previously described [22].

### SPM analysis

For both tracers, whole-brain voxel-wise comparisons of SUV and SUVR images between groups of P301S and WT mice were performed by SPM using SPM8 routines (Wellcome Department of Cognitive Neurology, London, UK) as previously established in our group [9]. This analysis was implemented in MATLAB (version 7.1), as adapted from Sawiak et al. [23] for mouse data. We performed two-sample *t* tests, setting a significance threshold of *p* < 0.05, uncorrected for multiple comparisons.

### Behavioral testing

*n* = 22 P301S and *n* = 18 WT mice were subjected to a MWM test for spatial learning and memory deficits, which was performed according to a standard protocol [20]. On training days one through five, each mouse had to perform four trials per day in the test basin, with maximum time set to 70 s. The test trial was performed on day six. For analyses of escape latency and distance during MWM testing, we used the video tracking software EthoVision® XT 13 (Noldus).

### Immunohistochemistry

A standard free-floating immunofluorescence (IF) protocol was performed in the cortex and brainstem areas matching the μPET brain regions. As previously described [9, 17], fixed 20-μm thick brain sections were first rinsed either overnight or for 48 h in PBS with 0.2% Triton X-100 containing one following primary antibodies: rabbit monoclonal IBA1 (1:500. Wako:19-19741), mouse monoclonal phospho-Tau (Ser202, Thr2015 (AT8), 1:500. Thermofisher: MN1020), and rat monoclonal CD68 (1:500. Bio-rad: MCA1857). After washing in PBS, sections were then incubated in a combination of three secondary antibodies (Alexa 488 goat anti-rabbit, Alexa 594 goat anti-mouse and Alexa 647 goat anti-rat IgG). For long-term preservation, the labelled slices were mounted in DAKO fluorescence mounting medium. Analyses for overall density (OD) and area coverage (area-%) were carried out by using the free access program ImageJ (https://imagej.nih.gov/ij/). The measurements were performed by using images obtained from a confocal microscope (LSM 780 Axio invers). Acquisitions were performed at × 20 and × 40 objectives in at least three sagittal sections. The implemented threshold tool was used for to limit the extracted data to the specific signal of the primary antibodies (IBA1, CD68, or AT8). To subtract unspecific signal from the OD, background staining signal, as obtained from immuno-negative regions, was deducted. OD per region was calculated for IBA1 and CD68 whereas the area-% was calculated for AT8.

### Statistics and calculations

Statistical analyses were performed in SPSS (Version 25, IBM Deutschland GmbH, Ehningen, Germany). The Kolmogorov-Smirnov test served to evaluate normal distribution of all data. Coefficients of variation (CoVs) were calculated as a measure of robustness in group data.

Two-tailed unpaired *t* tests were used to compare intergroup readouts (^18^F-FDG μPET, MWM, and IHC) of P301S and age-matched WT mice for all normally distributed readouts. For intergroup comparison of non-normally distributed readouts, Mann-Whitney *U* tests were calculated. Significance levels for intergroup comparison of longitudinal TSPO μPET were calculated by a mixed model repeated measures ANOVA. Effect sizes between P301S and WT were determined as Cohen’s *d*. For correlation analyses, Pearson’s coefficients of correlation (*R*) were calculated for normally distributed readouts. For non-normally distributed readouts, Spearman’s coefficients of correlation (*r*_S_) were calculated. Cortical TSPO μPET values in P301S were transformed into *z*-scores relative to WT findings as described previously and then plotted as a function of age. Our historical cortical TSPO μPET data of APP/PS1 (*n* = 17) [19] and *App*^*NL-G-F*^ (*n* = 21) [20] mice were reprocessed in the same way. Staining intensity as a function of age was likewise calculated for the current AT8 data in P301S mice and earlier findings of ^18^F-florbetaben *z*-score data in two historical amyloid-β mouse models. For TSPO μPET, we calculated the AUC of *z*-scores for all mice with successful completion of three or more serial μPET scans. AUC values were adjusted for the maximum *z*-score in the studied period, normalized to the observation time, and compared between tau and amyloid-β mice by ANOVA with Tukey post hoc correction. A validation analysis was performed for AUC within the same age range (2–7 months) of all three models. Division by the final TSPO *z*-score was omitted in this analysis due to limited serial data points. A threshold of *p* < 0.05 was considered to be significant for rejection of the null hypothesis.

## Results

### TSPO μPET facilitates monitoring of microglial activation in P301S mice

We first established that microglial activation can be monitored by longitudinal TSPO μPET imaging in P301S mice. To obtain robust TSPO μPET measures, we validated a suitable pseudo reference tissue: among the various possible regions, SUVR scaled TSPO μPET values in the nucleus accumbens (SUVR_NAC_) showed more robust group results when compared to conventional SUV scaling, as indicated by lower CoVs. For instance, SUVR_NAC_ CoVs in the brainstem were 6 ± 1% (range, 4 to 7%) whereas the corresponding SUV CoVs were 21 ± 3%; (range, 16 to 27%; Fig. 1d).

**Fig. 2:**
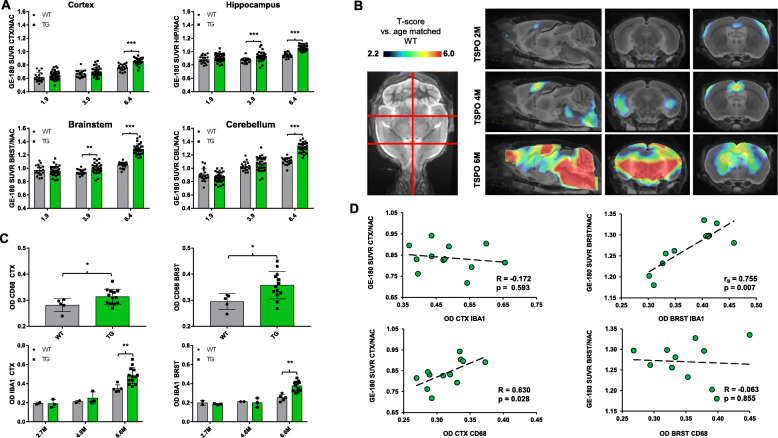
Longitudinal results of TSPO μPET imaging and immunohistochemistry validation. **a** Age dependent exponential increase of 18 kDa translocator protein (TSPO) expression in different target regions of the brain of P301S tau model mice. *n*_(P301S/WT)_ = 1.9 M, 33/18; 3.9 M, 32/17; 6.4 M, 29/17 (Mixed Model RM Anova of P301S vs wild-type per age). **b** Voxel-wise SPM analysis of TSPO expression in the contrast of P301S vs wild-type mice at different ages. *T*-score maps are projected upon an MRI template in sagittal and coronal slices. *n* equals to **a**. **c** Upper row: CD68 immunohistochemistry for the cortex and the brainstem of P301S mice and WT mice at 6.6 M. *n*_(P301S/WT)_ = 13/5. Lower row: Time dependent increase of IBA1 immunohistochemistry for the cortex and the brainstem of P301S mice and WT mice. *n*_(P301S/WT)_ = 2.7 M, 3/2; 4.8 M, 3/2; 6.6 M, 13/5. **d** Correlation plots of IBA1**/**CD68 immunohistochemistry and TSPO μPET at 6.4 months of age. *n*_(CTX)_ = 12, *n*_(BRST)_ = 11. M, months; OD, overall density; *R*, Pearson’s correlation coefficient; *r*_S_, Spearman’s correlation coefficient; CTX, cortex; BRST, brainstem; TG, transgenic P301S mice; WT, wild-type; *p < 0.05; **p < 0.01; ***p < 0.001. All error bars indicate SD

^18^F-GE-180 SUVR indicated exponential increases with age for all four target regions in P301S mice and significantly weaker comparable increases in WT mice. At 6.4 months, where was significantly elevated TSPO μPET signal in the cortex (+ 12%, *p* = 1.0E^−6^, Cohen’s *d* = 1.84), the hippocampus (+ 11%, *p* = 5.2E^−11^, Cohen’s *d* = 2.85), the brainstem (+ 23%, *p* = 3.4E^−14^, Cohen’s *d* = 3.74), and the cerebellum (+ 18%, *p* = 8.6E^−13^, Cohen’s *d* = 3.25) of P301S mice when compared to WT (Fig. 2a). Voxel-wise analyses at 6.4 months mirrored this finding for ^18^F-GE-180, with the most pronounced elevation in the hindbrain of P301S mice when compared to WT. However, SPM analysis revealed the first onset of increased ^18^F-GE-180 SUVR in P301S mice at 3.9 months of age (Fig. 2b). IHC with the microglial markers IBA1 and CD68 validated in vivo findings by indicating a similar increase of microglial activation with age in P301S mice when compared to increases of TSPO PET. Differences between P301S and WT mice at 6.6–6.7 months of age in IHC were also similar to TSPO μPET for the cortex (IBA1: + 39%, *p* = 0.011, Cohen’s *d* = 2.16/CD68: + 12%, *p* = 0.043, Cohen’s *d* = 1.29; Fig. 2c) and brainstem (IBA1: + 50%, *p* = 1.7E^−4^, Cohen’s *d* = 3.12/CD68: + 21%, *p* = 0.023, Cohen’s *d* = 1.56). The TSPO μPET signal in the cortex correlated significantly with the IHC for the phagocytosis marker CD68 (*R* = 0.630, *p* = 0.028), but not with IBA1 (*R* = − 0.172, *p* = 0.593) as a general marker of microglial activation, whereas the TSPO μPET signal in the brainstem correlated with IBA1 (*r*_S_ = 0.755, *p* = 0.007) but not with CD68 (*R* = − 0.063, *p* = 0.854; Fig. 2d). Table 1 provides a summary of TSPO μPET and microglia IHC results. Supplemental Figure 1 shows IBA1 and CD68 staining at different ages.

**Table 1:** Overview on μPET and immunohistochemistry (IHC) results

**Mouse strain**	**Age (months)**	**PET (*n*)**	**TSPO CTX**	**TSPO HIP**	**TSPO BRST**	**TSPO CBL**	**Age (months)**	**IHC (*n*)**	**IBA1 CTX**	**IBA1 BRST**	**CD 68 CTX**	**CD68 BRST**	**AT8 CTX**	**AT8 BRST**
**P301S**	1.9	33	0.64 ± 0.07	0.90 ± 0.06	0.96 ± 0.07	0.86 ± 0.07	2.7	3	0.19 ± 0.04	0.18 ± 0.01	**–**	**–**	2.63 ± 0.76	4.12 ± 0.12
	3.9	32	0.70 ± 0.07	0.93 ± 0.07***	0.99 ± 0.07**	1.06 ± 0.10	4.8	3	0.25 ± 0.05	0.20 ± 0.04	**–**	**–**	3.70 ± 0.89	4.56 ± 0.04
	6.4	29	0.85 ± 0.05***	1.05 ± 0.04***	1.28 ± 0.08***	1.31 ± 0.07***	6.6	14	0.49 ± 0.08**	0.38 ± 0.05**	0.31 ± 0.03*	0.36 ± 0.05*	5.57 ± 1.27	5.50 ± 0.95
**WT**	2.1	18	0.62 ± 0.07	0.88 ± 0.06	0.97 ± 0.07	0.90 ± 0.09	2.7	2	0.19 ± 0.01	0.20 ± 0.02	**–**	**–**	**–**	**–**
	4.1	17	0.67 ± 0.05	0.87 ± 0.04	0.94 ± 0.04	1.03 ± 0.07	4.5	2	0.21 ± 0.01	0.21 ± 3E-4	**–**	**–**	**–**	**–**
	6.5	17	0.76 ± 0.05	0.95 ± 0.03	1.05 ± 0.04	1.11 ± 0.05	6.7	5	0.35 ± 0.04	0.25 ± 0.03	0.28 ± 0.02	0.29 ± 0.03	0	0
**P301S**	**Age (months)**	**PET (*n*)**	**FDG CTX**	**FDG HIP**	**FDG BRST**	**FDG CBL**								
	6.4	24	0.78 ± 0.05	0.90 ± 0.03***	1.17 ± 0.04**	1.03 ± 0.05								
**WT**	6.1	16	0.75 ± 0.05	0.95 ± 0.05	1.22 ± 0.05	1.04 ± 0.05								

PET values are reported as SUVRNAC. *p* values were calculated by two-way *t* test or Mann Whitney *U* test of P301S vs age-matched wild-type (WT) mice

*FDG*^18^F-Fluorodesoxyglucose, *CTX* cortex, *HIP* hippocampus, *BRST* brainstem, *CBL* cerebellum

*p < 0.05, **p < 0.01, ***p < 0.001

### Longitudinal microglial response in P301S mice is attenuated and delayed when compared to amyloid-β mouse models

**Fig. 3:**
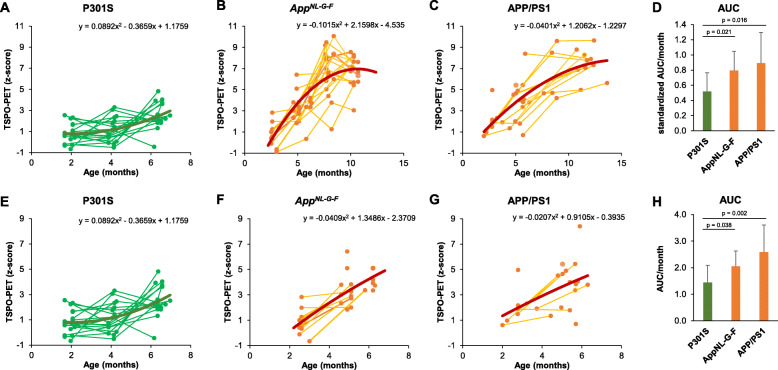
Standardized comparison of time courses of microglial activation between tau and amyloid-β mouse models. **a**–**c** Longitudinal TSPO expression in the cortex of tau and amyloid-β mouse models presented as *z*-score values against WT plotted with fitting as quadratic functions of age. **d** Standardized time courses of longitudinal TSPO expression in the cortex as expressed by the area under the curve (AUC) in three different mouse models. AUC values were normalized to the observation period and scaled by the *z*-score of the latest time point to control for absolute differences in TSPO expression of individual mice. **e**–**h** Validation analysis in similar age ranges across all three models. AUC values of the validation analysis (**h**) were normalized to the observation period but not to the latest time point. P301S: *n* = 18 mice, 52 data points; *App*^*NL-G-F*^: *n* = 21 mice, 68 data points; APP/PS1: *n* = 17 mice, 39 data points. Error bars of **d** and **h** indicate SD

Next, we asked if there are differences in the longitudinal development of microglial activation between the present tau mice and earlier studies in amyloid-β mouse models. To this end, we calculated standardized differences of TSPO μPET in the cortex of P301S, APP/PS1, and *App*^*NL-G-F*^ mice in comparison to WT and compared TSPO μPET as a function of age between models. Standardized differences were lower in P301S mice when compared to both amyloid-β mouse models (Fig. 3a–c). Longitudinal cortical TSPO μPET increases in the P301S mice followed a convex quadratic function (*y* = 0.09*x*^2^ − 0.37*x* + 1.18) whereas cortical TSPO μPET increases in amyloid-β mouse models were characterized by concave quadratic functions (*App*^*NL-G-F*^: *y* = − 0.10*x*^2^ + 2.16*x* − 4.53; APP/PS1: *y* = − 0.04*x*^2^ + 1.21*x* − 1.23; Fig. 3a–c). The TSPO μPET AUCs integrated over time were significantly higher in amyloid-β mouse models (*App*^*NL-G-F*^, 0.79 ± 0.25; APP/PS1, 0.89 ± 0.41) when compared to P301S mice (0.52 ± 0.25; *p* = 0.021/*p* = 0.016) after adjustment for the maximum increase at the ultimate age and normalization to the observation period (Fig. 3d). Importantly, there were concave quadratic increases over time for both tau to AT8 staining in P301S (*y* = − 0.10*x*^2^ + 1.69*x* − 1.29) and fibrillar Aβ to ^18^F-florbetaben μPET imaging in APP/PS1 (*y* = − 0.04*x*^2^ + 1.14*x* − 3.22) and *App*^*NL-G-F*^ (*y* = − 0.04*x*^2^ + 0.86*x* − 2.67) mice. The results of the full observation periods were validated by a direct comparison of similar age ranges between all models (Fig. 3e–h).

### Longitudinal microglial activation is associated with poor outcome parameters in P301S mice

**Fig. 4:**
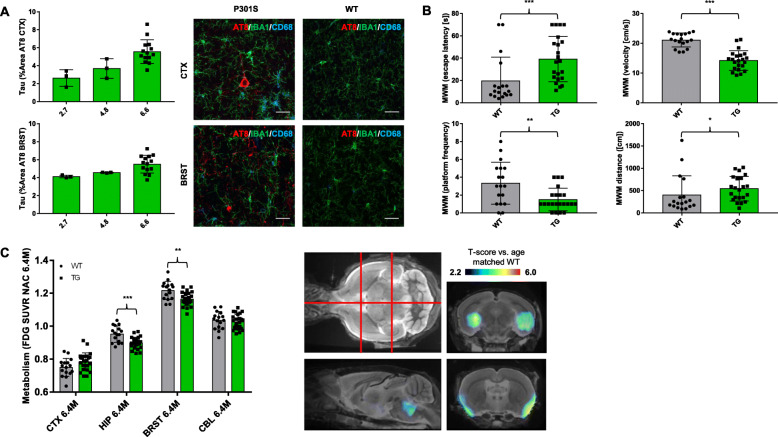
Outcome parameter in comparison of P301S and wild-type mice. **a** Longitudinal accumulation of tau measured via AT8 immunohistochemistry and representative images of co-staining with IBA1 and CD68 in the cortex (upper row) and brainstem (lower row). *n* (2.7 M/4.8 M) = 3; *n* (6.6 M) = 14. Scale bar = 30 μm. **b** Performance of mice in Morris water maze (MWM) at study termination with escape latency, velocity, swimming distance, and frequency of platform crossing. *n* = 22 P301S, *n* = 18 WT. **c** Glucose metabolism (FDG-PET uptake) in the different target VOIs at 6.4 months of age and SPM results of the contrast P301S and wild-type (WT) mice. *n* = 24 P301S, *n* = 16 WT. Color coding shows regions with decreased glucose metabolism (*T*-scores) in P301S mice when compared to WT upon an MRI template in sagittal and coronal slices, as indicated by red lines in the axial MRI slice. CTX, cortex; HIP, hippocampus; BRST, brainstem; CBL, cerebellum; M, months; SUVR, standardized uptake value ratio; *p < 0.05; **p < 0.01; ***p < 0.001. All error bars indicate SD

Finally, we endeavored to test if baseline or longitudinal TSPO μPET can predict neuropathological, behavioral, and functional outcome measures in P301S mice. To this end, we recorded tau quantification, a spatial learning paradigm, and ^18^F-FDG μPET at the terminal time-point and correlated these three endpoints with baseline and longitudinal TSPO μPET readouts. AT8 positive tau accumulation was confirmed by immunohistochemistry, which showed regional colocalization with activated microglia in the cortex and the brainstem (Fig. 4a). P301S mice aged 6.3 months took twice as long as WT mice to find the virtual platform in MWM (P301S 39 ± 20 s; WT 20 ± 21 s; *p* = 3.4E^−4^) and revealed a lower frequency of crossing the platform area (P301S 1.5 ± 1.2 times, WT 3.3 ± 2.3 times; *p* = 0.010). Swim speed of WT mice was significantly faster when compared to P301S mice (21 ± 2 cm/s vs 14 ± 3 cm/s, *p* = 4.8E^−8^). Therefore, the travelled distance was additonally analyzed as a readout unbiased from locomotor problems. Significantly longer travelled distance in P301S mice when compared to WT (544 ± 268 cm vs 404 ± 414 cm, *p* = 0.019) validated the deficits in spatial memory independent from beginning locomotor problems (Fig. 4b). ^18^F-FDG μPET revealed significantly decreased glucose metabolism of P301S mice compared with WT mice at 6.4–6.5 months of age in the hippocampus (− 6%; *p* = 1.9E^−4^) and the brainstem (− 4%; *p* = 0.002), which was also mirrored by voxel-wise analyses (Fig. 4c).

**Fig. 5:**
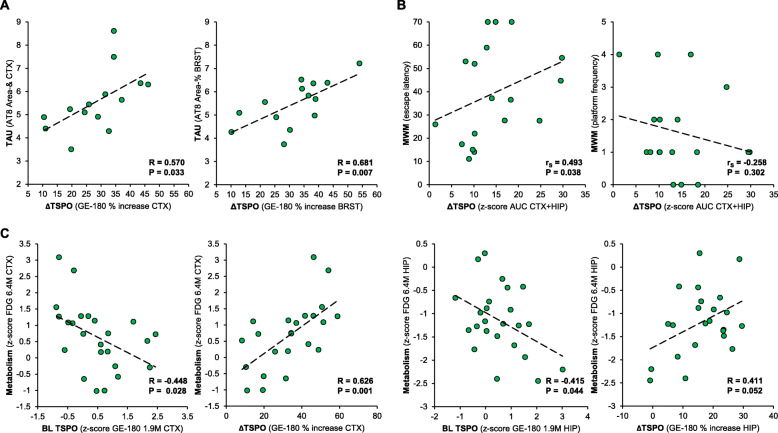
Associations of early and longitudinal microglial activation with outcome parameters. **a** Correlation of longitudinal TSPO expression in P301S mice (expressed as %-increase between 1.9 and 6.4 months of age) with accumulation of AT8 positive tau accumulation in IHC for the cortex and the brainstem; *n* = 14. **b** Correlation of combined longitudinal TSPO expression in cortex and hippocampus (calculated as AUC) with terminal MWM performance; *n* = 18. **c** Correlations of early and longitudinal TSPO expression (BL *z*-score and %-increase) with terminal glucose metabolism (FDG PET *z*-score) for the cortex and the hippocampus; *n* = 24

Longitudinal increases of the TSPO μPET signal were positively correlated with AT8 accumulation in the cortex (*R* = 0.570, *p* =0.033) and in the brainstem (*R* = 0.681, *p* = 0.007, Fig. 5a). Baseline TSPO μPET did not significantly predict tau accumulation.

Higher TSPO μPET values over time in the cortex and the hippocampus of P301S mice were associated with worse performance in MWM at the late stage (escape latency: *r*_S_ = 0.493; *p* = 0.038; frequency: *r*_S_ = − 0.258, *p* = 0.302; Fig. 5b). Baseline TSPO μPET did not significantly predict MWM readouts at 6.3 months of age.

Baseline TSPO μPET elevation in the cortex (*R* = − 0.448; *p* = 0.028) and in the hippocampus (*R* = − 0.415; *p* = 0.044) of P301S mice at 1.9 months of age predicted stronger hypometabolism in FDG μPET at 6.4–6.5 months of age (Fig. 5c). On the other hand, longitudinal increases of the TSPO μPET signal in these regions were associated with elevated glucose metabolism or predicted a trend towards elevated glucose metabolism at 6.4–6.5 months of age (Fig. 5c).

## Discussion

We report the first longitudinal in vivo μPET imaging study of microglial activation together with assessment of multiple outcome parameters in a tau mouse model. Our data clearly indicate that μPET with the TSPO tracer ^18^F-GE-180 gives reliable assessment of microglial activation in living P301S mice, as proven by the high correlation with specific IHC markers. Analysis of individual TSPO μPET time courses to 6.4 months of age revealed that microglial activation in the tau model mice exponentially increases with age. Importantly, longitudinal elevations of TSPO expression in P301S mice predicted aggravated tau accumulation and worse performance in spatial learning. Levels of glucose metabolism at the late stage were positively associated with longitudinal TSPO μPET increases, but early elevations of TSPO expression predicted stronger hypometabolism in P301S mice. These findings may be reconciled by consideration of the ambivalent role of microgliosis in neurodegeneration, in some circumstances imparting neuroprotection, and in other circumstances marking a more aggressive pathology. This dual role is seemingly decided by the type of pathology (i.e., tau or amyloid-β over-expression) and the time course.

We show that transfer of TSPO μPET technology from different amyloid-β mouse models [19, 22, 24] to the present tau mouse model is feasible without major caveats. As in some former studies [16, 20], we successfully validated a suitable pseudo reference region for TSPO μPET in P301S mice, and we were again able to show that this methodology reduces variance at the group level. This SUVR approach supported the detection of robust longitudinal increases of TSPO expression in different target regions of the P301S mouse model, which were matched with increases of microglial activation markers measured later by IHC. Furthermore, our μPET data were validated by direct correlation with IHC at the terminal time point. Interestingly, the phagocytosis marker CD68, which indicated the highest association with TSPO μPET in our earlier study of amyloid-β mice [19], significantly correlated with TSPO μPET values in the cortex but not in the brainstem of tau mice. Yet, the present correlation of ^18^F-GE-180 TSPO μPET with the more general activation marker IBA1 in the brainstem of tau mice could hinge on different microglia phenotypes and their covariance with TSPO expression in different brain regions [25]. However, we note that several specific and random factors like regional blood flow or slice selection could also cause these divergent observations. Our findings are in line with those of a study conducting longitudinal μPET with the TSPO tracer ^11^C-AC-5216 in another tau mouse model (PS19), likewise showing time dependent progression of TSPO expression in the entorhinal cortex and the hippocampus [26]. Another study of PS19 mice found microglial activation especially in the hippocampus to occur ahead of discernible tau accumulation and brain atrophy [27]. This implicates that microglial activation can already be present in very early phases of AD, when seeding of tau just begins and is not identifiable by tau IHC yet. This also suggests that neuroinflammation could be triggered by non fibrillar components of tau, ending up in a vicious circle in the pathogenesis of AD. However, we note that many of the complex underlying mechanisms and interrelations are not completely discovered yet [3, 5, 28]. Understanding the role of neuroinflammation in neurodegenerative disorders is of high importance as it is associated with deposition of NFT, amyloid-β, and neuronal degeneration. Translational imaging in mouse models and patients will facilitate to close important research gaps in terms of reciprocal validation and therapy monitoring.

As there have been no direct comparisons of the time courses of microglial activation between amyloid-β and tau mouse models, we put a special focus on contrasting longitudinal in vivo TSPO expression of P301S mice against existing data in two common amyloid-β mouse models. To account for natural progression of microglial activation in the aging brain of rodents [17], we compared standardized differences (*z*-scores) in relation to age among the different mouse models. By this approach, we are able to show for the first time that temporal kinetics of microglial activation differ depending on whether it is driven by tau or amyloid-β pathology. In particular, TSPO expression in response to tau pathology showed attenuated and delayed development when compared to TSPO expression in response to Aβ overexpression. Importantly, there were similar increases of the amounts of AT8 positive tau in P301S mice or fibrillar Aβ in APP/PS1 and *App*^*NL-G-F*^ mice with age, indicating that the observed differences were not driven by variant time courses of protein accumulation. In the translational aspect, the onset of Aβ and tau aggregation may precede the start of clinical symptoms in human AD [29], and associations of both proteins with microglial activation have already been shown in human PET studies [6]. Thus, the presence of amyloid-β and tau should be considered (i.e., by PET or CSF) when interpreting time courses of microglial activation in human neurodegenerative disease, to avoid bias arising from differences in their temporal associations. Furthermore, more detailed studies comparing tau and amyloid-β mouse models employing next generation sequencing or proteomics should resolve possible differences of microglia phenotypes in relation to the two abnormal protein aggregates.

Details of the role and time dependence of neuroinflammation in AD remain a matter of controversy and debate, given the ambivalence of protective and detrimental aspects [3, 28, 30]. This also accounts for some earlier findings on microglial function in the P301S mouse model. Luo et al. [14] showed in vitro and ex vivo the capability of isolated wild-type microglia to phagocytize tau in brain tissue of P301S mice, implicating a possible protective effect of fully functional microglia. On the other hand, a recent study of Brelstaff et al. [15] indicated that activated microglia can phagocytize neurons of P301S mice and therefore seemingly mediate deleterious effects. This matches with the setting that microglial cells can generally be separated into two classes, proinflammatory (mainly detrimental) and anti-inflammatory (mainly protective) phenotypes, and can presumably change from one state to another depending on the required function [28].

The strength of our data lies in its longitudinal in vivo design, covering approximately three quarters of the 9-month lifespan of P301S model mice. The compilation of data indicates that higher longitudinal TSPO expression in P301S mice predict higher tau accumulation and worse spatial learning at 6.4 months of age. Interestingly, the corresponding associations with glucose metabolism to ^18^F-FDG μPET gave different predictions for baseline and longitudinal measures. While early elevation in TSPO expression was associated with hypometabolism at 6.4 months, we observed higher terminal glucose metabolism in tau mice with TSPO increasing over time. While the first result suggests an overall deleterious effect of high early TSPO expression on the outcome of P301S mice, the second observation is more consistent with a coupling of microglial activation and glucose metabolism, as observed previously in PS2APP mice [16]. With regard to spatial learning deficits, our earlier study with congruent methodology and design in PS2APP amyloid-β mice showed that early microglial activation in the forebrain strongly correlated with better cognitive performance in MWM [31]. Speculatively, this could indicate different predictive capability of TSPO μPET depending on whether tau or amyloid-β accumulation is the primary driver of microgliosis. Regarding tau mouse models, our observation of higher tau accumulation in mice with early microglial activation is in line with findings of associated tau and neuroinflammation in the forebrain of rTg4510 tau mice [32]. Our data also fit with the observations of attenuated NFT accumulation, reduced neuronal degeneration, and averted cognitive deterioration after pharmacological ablation of senescent microglial and astroglial cells in PS19 mice [33], as well as fitting with the increased tau pathology occurring along with NLRP3 inflammasome activation [7]. In summary, tau-associated microglial activation seems more detrimental than amyloid-β-associated effects. Importantly, our compilation of findings of amyloid-β and tau mouse models may help support a model wherein the net effect of neuroinflammation changes from being initially protective (A+/T−) to deleterious in late phases (A+/T+) of AD [34]. Among the limitations of our study, we note that we did not perform longitudinal TSPO-PET imaging in male mice. Thus, we cannot evaluate effects of sex on the current results. We acknowledge a gap of 1 week in average between the final PET scan and IHC which might has a limited impact on correlation analyses between both modalities since microglial activation in P301S mice raises with aging.

## Conclusions

Monitoring of microglial activation in P301S tau transgenic is feasible by μPET with the TSPO tracer ^18^F-GE-180 TSPO, as validated by IHC with microglial markers. P301S mice manifest delayed time courses and detrimental associations of their microglial activation with outcome parameters when compared to earlier data of amyloid-β mouse models. This should draw further attention to the study of phenotypic differences of the microglial responses to amyloid-β and tau accumulation.

## Supplementary information

**Additional file 1: Supplemental figure 1.** Overview of representative images of IBA1 and CD68 immunohistochemistry (IHC) stainings from 2.7-6.6 M: The upper row shows representative images of the cortex (CTX), the lower row shows representative images of the brainstem (BRST). Magnification 40x objective/oil; scale Bar: 30 μm; WT = wild-type; TG = P301S; M = age in months.

## Data Availability

The datasets used and/or analyzed during the current study are available from the corresponding author on reasonable request.

## Abbreviations

Aβ: Beta amyloid
AD: Alzheimer’s disease
AUC: Area under the curve
BRST: Brainstem
CBL: Cerebellum
CoVs: Coefficients of variation
CSF: Cerebrospinal fluid
CTX: Cortex
(^18^F-)FDG: ^18^F-fluordesoxyglucose
HIP: Hippocampus
IF: Immunofluorescence
IHC: Immunohistochemistry
MAPT: Microtubule-associated protein tau
μPET: Small animal positron-emission-tomography
MRI: Magnetic resonance imaging
MWM: Morris water maze
NAC: Nucleus accumbens
NFT: Neurofibrillary tangles
OD: Overall density
p.i.: Post injectionem
*R*: Pearson’s coefficient of correlation
*r*_s_: Spearman’s coefficient of correlation
SPM: Statistical parametric mapping
SUV: Standardized uptake value
SUVR: SUV-ratio
TSPO: Translocator protein
VOIs: Volumes of interest
WT: Wild-type
