# Supplementary figures and images for: Longitudinal TSPO expression in tau transgenic P301S mice predicts increased tau accumulation and deteriorated spatial learning
Source: J Neuroinflammation. 2020 Jul 13;17:208. doi: 10.1186/s12974-020-01883-5 (PMC7358201; doi:10.1186/s12974-020-01883-5)

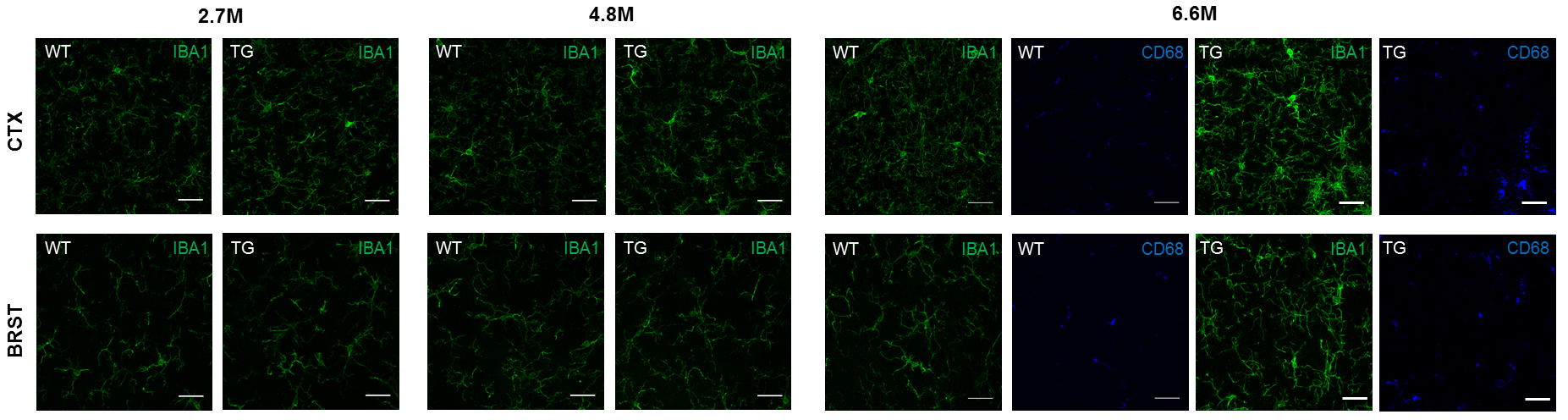

Supplement: Supplementary file 1 — Additional file 1: Supplemental figure 1. Overview of representative images of IBA1 and CD68 immunohistochemistry (IHC) stainings from 2.7-6.6 M: The upper row shows representative images of the cortex (CTX), the lower row shows representative images of the brainstem (BRST). Magnification 40x objective/oil; scale Bar: 30 μm; WT = wild-type; TG = P301S; M = age in months. [file 12974_2020_1883_MOESM1_ESM.docx]
